# Chronic oral safety study of the aqueous extract of *Combretum molle* twigs on biochemical, haematological and antioxidant parameters of Wistar rats

**DOI:** 10.1186/s12906-020-02896-6

**Published:** 2020-04-05

**Authors:** David Miaffo, Sylvie Léa Wansi, Fidèle Ntchapda, Albert Kamanyi

**Affiliations:** 1 Department of Life and Earth Sciences, Laboratory of Physiology, Higher Teachers’, Training College, University of Maroua, P.O. Box 55, Maroua, Cameroon; 2 Department of Animal Biology, Laboratory of Animal Physiology and Phytopharmacology, Faculty of Science, University of Dschang, P.O. Box 67, Dschang, Cameroon; 3 Department of Biological Sciences, Faculty of Science, University of Ngaoundéré, P.O. Box 454, Ngaoundéré, Cameroon

**Keywords:** Chronic toxicity, *Combretum molle*, Wistar rats, Biochemical parameters, Haematological parameters, Antioxidant, Aqueous extract

## Abstract

**Background:**

*Combretum molle* R.B/G. Don (Combretaceae) is a graceful deciduous shrub, distributed especially in tropical Africa and used in traditional medicine in the treatment of malaria, diabetes, and bacterial, liver and cardiovascular deseases. To our knowledge, no long-term toxicity studies of *C. molle* has ever been realized yet.

**Methods:**

The long-term toxicity study was conducted in accordance with OECD 408 guidelines with slight modifications. In fact, rats were divided in groups and treated orally with CMAE at doses of 62.5, 125 and 250 mg/kg for 6 months. The general behavior and signs of toxicity of the rats were daily observed. Body weight, food and water intake were recorded every 2 months for 6 months. At the end of treatment period, urine and blood samples were collected for hematological, biochemical and antioxidant estimations. Immediately, internal organs were collected and weighed.

**Results:**

The results showed that no mortality and visible signs of the toxicity were recorded in all experimental animals. The administration of CMAE had no significant effects on body weight, organ weights, serum electrolyte, and food and water intake. However, all doses of CMAE produced an increase in high density lipoprotein cholesterol, white blood cells, platelets, glutathione, and a decrease in low density lipoprotein cholesterol and malondialdehyde rate. CMAE at doses of 125 and 250 mg/kg decreased in serum proteins and the activity of aspartate amino transferase, and increased the activity of catalase. In addition, CMAE (250 mg/kg) significantly decreased the alanine aminotransferase activity and the level of triglycerides, very low density cholesterol, total proteins and creatinine, and increased in renal clearance, red blood cells, hemoglobin, hematocrit and superoxide dismutase activity.

**Conclusions:**

At the end of this study, no signs of major intoxication was noted during 6 months of treatment. These results suggest that long-term consumption of CMAE at the therapeutic dose (250 mg/kg) presents low risks to human health.

## Background

The use of medicinal plants is widespread and of increasing health and economic importance. In Africa and Asia, up to 80% of the population uses traditional medicine to meet their health care needs [1]*.* In developed countries where the use of modern medicine is predominant, traditional medicine is becoming more and more popular [2]. For rational use of folk pharmacopoeia, much research is being done on traditional recipes to obtain improved traditional medicines (MTA). However any biologically active substance is likely to produce in the short, medium or long term undesirable or even harmful effects. It is therefore necessary to carry out a toxicological study of each substance in order to evaluate the risks it poses to any living being following a voluntary or accidental exposure.

*Combretum molle* RB/G.Don (Combretaceae) called Djokirdowal in the Far North of Cameroon is a graceful deciduous shrub, measuring 3 to 13 m in height, which idwidespread in tropical Africa [3]. It is used in African medicine for the treatment of many diseases. In Mali, leaves of *C. molle* are used to treat diarrhea, paronychia and stomach upset. In Zimbabwe, root decoctions are used for difficult births. In Kenya, roots also help prevent circulatory diseases. In Tanzania, the powder of dried roots is applied to stop vaginal candidiasis [4]. In Cameroon, a decoction of twigs, leaves and barks of *C. molle* is used to treat diabetes, inflammation and bacterial diseases, respectively. This plant has a long been proven effective in the treatment of liver disease, malaria, tuberculosis, snake bites and general body swelling [5].

The results of the phytochemical screening revealed that CMAE twigs contains carbohydrates, proteins, tannins, phenols, flavonoids, coumarins, quinones, saponins, terpenoids and phlobatannins [6]. *C. molle* also contains two types of triterpenes (3β, 7α-dihydroxy-28-hydroxy-methyl-lanost-5-ene and 3β-hydroxy-28-hydroxy-methyl-lanost-25 (26)-ene and 1α-hydroxy cycloartenide (mollic acid) [7].

Previous pharmacological studies have shown that the *C. molle* has antihypertensive, antihelminthic, diuretic, antibiotic and cytotoxic effects [7]. However, the presence of glucidomollic acid and 1α-hydroxycycloartenoid confers it to play an analgesic and anti-inflammatory role [8]. Simon et al. [9] reported that the methanol extract of *C. molle* barks has antibacterial, antifungal and anthelmintic effects. Ojewole and Adewole [10] findings confirmed the hypoglycemic, anti-inflammatory, analgesic and cardiovascular effect of glycosides extracted from leaves of *C. molle*. The antidiabetic effects of *C. molle* twigs have been proven by Miaffo et al. [6, 11].

Toxicological studies have shown that the aqueous extract of leaves and twigs *C. molle* was weakly toxic at the dose of 2000 mg/kg [11, 12]. The medium-term administration of extracts of *C. molle* twigs at the dose of 500 mg/kg resulted in the loss of appetite, growth disorders and light tissue damage [13]. However, no long-term toxicity studies of *C. molle* has ever been, realized yet. The aim of the present work was therefore to evaluate the chronic toxicity of the aqueous extract of *C. molle* twigs in rats.

## Methods

### Plant collection and identification

The fresh twigs of *C. molle* were harvested in August 2014 in Moutourwa (Far North Region, Cameroon), identified and authenticated by Mr. Victor Nana, botanist at the National Herbarium of Cameroon (NHC), where the voucher specimen was deposited (number 433724HNC). The twigs of *C. molle* were chopped into small pieces, washed with tap water and shade dried. Upon complete desiccation, the dried material was reduced into a powder using a grinding mill.

### Preparation of plant extract

Two hundred grams (200 g) of *C. molle* powder were added to 500 ml of distilled water and the whole system was boiled for 15 min. After cooling, the mixture was filtered using Wattman no1 filter paper. The filtrate was evaporated in an oven at 45 °C in order to obtain 19.69 g of crude extract, ie a yield of 9.84%. The crude extract was then stored at 4 °C and dissolved in distilled water prior to administration.

### Animals

Wistar rats of both sexes (males and females) aged 8 to 10 weeks and weighing 120 to 140 g were purchased from the animal house of the Laboratory of Animal Physiology, Department of Biology and Animal Physiology of the University of Yaoundé I. They were kept in polypropylene cages, under conditions of ambient temperature (24–26 °C) and natural brightness (12 h light /12 h dark cycle). Throughout the experiment, all animals had free access to the water and standard diet. Animals were acclimatized in laboratory conditions 7 days before the beginning of the experiment. All animal experiments were handled according to the Cameroon National Ethics Committee (Ref. N° FWIRB 00001954) and all experiments have been examined and approved.

### Experimental design

The long-term toxicity study was conducted in accordance with OECD 408 guidelines [14] with slight modifications linked to the WHO guidelines [15], where the different treatments are administered orally to rodents. The daily amount of *C. molle* that the traditional healer gives to adult patients is 2835 mg. This mass of extract supposedly consumed by an adult of 70 kg allowed us to calculate the human therapeutic dose which is 40.5 mg/kg. The equivalent dose in rats was approximately 250 mg/kg, calculated according to the formula of Shannon et al. [16]. The doses of 62.5, 125 and 250 mg/kg were used for this test.

### Distribution and treatment of animals

Forty (40) rats were divided into 4 groups of 10 rats each (5 males and 5 females) and treated by gavage as follows:

- Group 1 (control group) received distilled water for 6 months.

- Group 2 received 62.5 mg/kg bw of CMAE for 6 months.

- Group 3 received 125 mg/kg bw of CMAE for 6 months.

- Group 4 received 250 mg/kg bw of CMAE for 6 months.

After the administration of the various treatments at the last day of the experience, the animals were individually placed in metabolic cages where they received drinking water. Twenty-four hour later, urine was collected and stored at − 20 °C for the assay of biochemical parameters. Immediately after urine collection, all animals were sacrificed by cervical decapitation under anesthesia (10 mg/kg b.wt diazepam and 50 mg/kg b.wt. ketamine i.p.) and the abdominal cavity was opened. Blood samples were collected (by cardiac puncture) in the heparinized tubes, and in dry tubes without anticoagulant. The blood under anticoagulant was used directly to evaluate the hematological parameters while non-heparinized blood was left for 1 h at rest for coagulation and then centrifuged (Searchtech 92–2) at 3000 rpm for 15 min. The resulting supernatant (serum) after centrifugation was stored at − 20 °C for the determination of biochemical and antioxidant parameters.

### Mortality and clinical signs of toxicity

Mortality, behavioral changes (mobility, aggressiveness, drooling, and sensitivity to pain, noise and touch) and signs of toxicity (stool condition, difficulty in breathing, convulsion, lethargy, coma and tumor) were evaluated throughout the duration of the experiment.

### Evaluation of body weight, relative weight of organs and food and water consumption

Body weight and food and water intake were assessed every 2 months for 6 months. Immediately after blood collection, internal organs such as liver, heart, kidneys, lungs, spleen, stomach, ovaries, testes and pancreas were collected, freed from fatty material cleaned in NaCl 0.9%, wrung and weighed.

### Hematological analysis

Hematological parameters were evaluated using an automatic hematological analyzer (Sysmex KX-21, Japan). These parameters include red blood cells (RBC), hematocrit (HCT), hemoglobin (Hb), mean corpuscular volume (MCV), mean corpuscular hemoglobin (MCH), mean corpuscular concentration in hemoglobin (MCCH), white blood cells (WBC), distribution index of red blood cells, neutrophils, monocytes, lymphocytes, eosinophils, basophils, platelets, mean platelet volume (MPV) and the platelet distribution index .

### Biochemical analysis

Glucose, total cholesterol, high density lipoprotein (HDL-c), low density lipoprotein (LDL-c), total proteins, total bilirubin, alanine aminotransferase (ALT), aspartate amino transferase (AST), alkaline phosphatase (ALP), urea, uric acid, sodium, potassium, phosphorus and chloride were determined using an automated analyzer. Very low density cholesterol (VLDL) was calculated by the following formula: VLDL = 0.2 × TG [17]. Creatinine was determined using alkaline picrate method described by Jaffe [18]. Renal clearance (CR) was calculated using the formula: CR = [creatinine] urine × 24 h urine volume ***/*** [creatinine] serum [19].

### Antioxidant analysis

Malondialdehyde (MDA) was evaluated according to the method of Draper and Hadley [20]. Glutathione (GSH) was assayed according to the method of Sehirli et al. [21]. Catalase (CAT) was assayed according to the method of Luck [22]. Superoxide dismutase (SOD) was determined by the method of Sun et al. [23].

### Statistical analysis

All results are expressed as mean ± SD (Standard Derivation). Statistical analysis were achieved using ANOVA followed by Turkey post test using Graph Pad Prism Software version 5.0. Statistical significance was considered at *p* < 0.05 values.

## Results

### Mortality and clinical signs of toxicity

During the 6 months of experiment, no mortality and visible signs of the toxicity were recorded in the animals of the control group and those of the groups that received the different doses of plant extract.

### Body weight and relative organ weights

The body weight and relative organ weights of animals treated with different doses of CMAE are shown in Tables 1 and 2, respectively. It show that, no significant difference (*p* > 0.05) of body weight and relative weight of internal organs of the CMAE treated subjects were noted during the entire treatment period, compared to the control group.

**Table 1:** Body weight of rats after 6 months of treatment with the aqueous extract of *C. molle*

Mean body weight (g)				
Group	Month 0	Month 2	Month 4	Month 6
Control	130.2 ± 1.81	143.0 ± 1.80	173.9 ± 1.97	192.2 ± 1.49
62.5 mg/kg	128.6 ± 1.48	147.2 ± 2.31	167.6 ± 1.41	195.0 ± 2.33
125 mg/kg	132.1 ± 1.57	152.8 ± 2.85	184.3 ± 3.27	203.2 ± 5.03
250 mg/kg	129.6 ± 1.73	149.9 ± 1.49	180.6 ± 1.78	196.4 ± 3.69

Values are expressed as mean ± SD, *n* = 10. (One-way ANOVA followed by Turkey’s post-hoc test)

**Table 2:** Relative organ weight of rats after 6 months of treatment with the aqueous extract of *C. molle*

Relative weight (g/100 g b.wt.)				
Organs	Control	62.5 mg/kg	125 mg/kg	250 mg/kg
Liver	3.446 ± 0.099	3.003 ± 0.092	3.099 ± 0.152	3.302 ± 0.147
Kidneys	0.760 ± 0.015	0.731 ± 0.021	0.687 ± 0.033	0.706 ± 0.025
Lungs	0.905 ± 0.022	0.834 ± 0.026	0.922 ± 0.022	0.828 ± 0.021
Heart	0.346 ± 0.016	0.317 ± 0.011	0.352 ± 0.015	0.334 ± 0.012
Spleen	0.218 ± 0.012	0.267 ± 0.020	0.208 ± 0.014	0.204 ± 0.010
Pancreas	0.309 ± 0.015	0.298 ± 0.015	0.273 ± 0.011	0.315 ± 0.011
Stomach	1.236 ± 0.026	1.206 ± 0.020	1.171 ± 0.041	1.193 ± 0.024
Adrenal glands	0.036 ± 0.001	0.034 ± 0.001	0.032 ± 0.001	0.035 ± 0.001
Testes	1.403 ± 0.024	1.366 ± 0.018	1.315 ± 0.040	1.338 ± 0.030
Ovaries	0.079 ± 0.003	0.085 ± 0.001	0.083 ± 0.004	0.086 ± 0.002

Values are expressed as mean ± SD, *n* = 10. (One-way ANOVA followed by Turkey’s post-hoc test)

### Food and water consumption

According to Tables 3 and 4, no significant variation in food and water consumption was recorded in rats treated with the different doses of CMAE as compared to those receiving distilled water.

**Table 3:** Food intake of rats after 6 months of treatment with the aqueous extract of *C. molle*

Food intake (g/day)				
Group	Month 0	Month 2	Month 4	Month 6
Control	7.4 ± 0.34	8.9 ± 1.09	9.5 ± 0.27	8.4 ± 0.37
62.5 mg/kg	8.9 ± 0.48	7.4 ± 0.45	9.6 ± 0.65	7.2 ± 0.29
125 mg/kg	8.7 ± 0.60	9.4 ± 0.54	10.9 ± 0.86	10.0 ± 0.39
250 mg/kg	9.8 ± 0.87	10.4 ± 1.39	12.0 ± 0.76	11.3 ± 0.70

Values are expressed as mean ± SD, *n* = 10. (One-way ANOVA followed by Turkey’s post-hoc test)

**Table 4:** Water intake of rats after 6 months of treatment with the aqueous extract of *C. molle*

Water intake (mL/day)				
Group	Month 0	Month 2	Month 4	Month 6
Control	15.4 ± 0.70	12.1 ± 1.00	14.1 ± 0.53	13.5 ± 0.48
62.5 mg/kg	18.2 ± 1.07	14.9 ± 1.08	16.5 ± 1.10	17.6 ± 0.78
125 mg/kg	17.5 ± 1.64	18.4 ± 1.98	14.1 ± 1.13	17.7 ± 0.60
250 mg/kg	18.6 ± 1.00	15.1 ± 0.62	18.3 ± 0.93	15.8 ± 0.57

Values are expressed as mean ± SD, *n* = 10. (One-way ANOVA followed by Turkey’s post-hoc test)

### Hematological parameters

Table 5 shows the effect of CMAE on some hematological parameters of rats after 6 months of treatment. It shows that no significant changes of parameters such as MCV, MCH, MCCH, MPV, monocytes, lymphocytes and granulocytes was recorded in subjects who received different doses of extract, compared to the control group. The number of WBC significantly increased in the animals treated at doses of 62.5 (*p* < 0.01), 125 (p < 0.01) and 250 mg/kg (*p* < 0.001) of extract. A significant increase in the rate of PLT (*P* < 0.001) was noted in animals treated with different doses of extract. In addition, the dose of 250 mg/kg produced a significant increase in the number of RBC (*p* < 0.01), Hb (p < 0.01) and HCT (*p* < 0.05).

**Table 5:** Some hematological parametres of rats after 6 months of treatment with the aqueous extract of *C. molle*

Parameters	Groups			
	Control	62.5 mg/kg	125 mg/kg	250 mg/kg
RBC (10^6^/ mm^3^)	7.37 ± 0.4	8.24 ± 0.30	8.4 ± 0.05	9.42 ± 0.23**
Hb (g/ dL)	12.77 ± 0.09	13.57 ± 0.53	13.53 ± 0.07	15.27 ± 0.31**
HCT (%)	42.7 ± 1.23	43.16 ± 0.47	44.6 ± 0.81	50.00 ± 2.49*
WBC (10^3^/mm^3^)	4.47 ± 0.14	6.37 ± 0.29**	6.57 ± 0.28**	6.90 ± 0.25***
PLT (10^3^/mm^3^)	276.00 ± 2,89	303.00 ± 4.17***	323.67 ± 4.17***	340.67 ± 3.84***
MCV (pg)	53.67 ± 0.89	52.14 ± 0.47	52.67 ± 1.46	57.67 ± 0.89
MCH (g/dL)	16.24 ± 0.14	16.34 ± 0.03	16.24 ± 0.30	16.43 ± 0.12
MCCH (g/dL)	29.50 ± 0.38	30.97 ± 0.38	31.00 ± 1.05	29.23 ± 0.96
MPV (fL)	11.03 ± 0.08	10.57 ± 0.21	8.77 ± 0.12	9.70 ± 0.17
Lymphocytes (%)	45.77 ± 0.27	50.17 ± 2.10	48.3 ± 1.20	47.63 ± 1.16
Monocytes (%)	10.90 ± 0.05	11.97 ± 0.24	11.00 ± 0.30	11.16 ± 0.59
Granulocytes (%)	27.87 ± 0.29	29.14 ± 1.01	27.30 ± 0.35	28.37 ± 1.21

Values are expressed as mean ± SD, *n* = 10. (One-way ANOVA followed by Turkey’s post-hoc test). *P < 0.05; **P < 0.01; ***P < 0.001 significantly different when compared to the control group

### Biochemical parameters

The effect of the decoction of *C. molle* twigs on the biological parameters of the animals is shown in Table 6. It can be seen from this table that no significant variation in blood glucose, total bilirubin, albumin, ALP, total cholesterol, urea, uric acid and urinary creatinine was recorded in all rats who received different doses of extract, compared to those in the control group.

**Table 6:** Some biochemical parametres of rats after 6 months of treatment with the aqueous extract of *C. molle*

Parameters	Groups			
	Control	62.5 mg/kg	125 mg/kg	250 mg/kg
Glucose (mg/dL)	82.2 ± 2.5	86.9 ± 2.3	79.7 ± 1.8	76.4 ± 2.4
T. protein (g/dL)	8.31 ± 0.65	7.42 ± 0.48	8.79 ± 0.28	9.18 ± 0.48
T. biliribin (μmol/L)	12.46 ± 0.93	13.30 ± 1.09	13.79 ± 1.21	14.24 ± 1.37
Albumin (g/L)	46.55 ± 2.93	41.53 ± 3.23	41.70 ± 3.93	39.14 ± 3.21
ALT (U/L)	98.60 ± 6.0	95.11 ± 10.16	103.75 ± 4,19	57.47 ± 8.33**
AST (U/L)	110.22 ± 4.90	97.18 ± 7.29	85.73 ± 5.01*	82.73 ± 7.19*
ALP (U/L)	93.37 ± 4.62	95.37 ± 4.85	83.38 ± 6.25	99.93 ± 4.08
Cholesterol (mg/dL)	99.24 ± 4.87	107.27 ± 3.02	109.40 ± 5.69	89.55 ± 1.83
Triglycerides (mg/dL)	75.82 ± 3.60	90.84 ± 5.42	84.92 ± 5.14	44.59 ± 4.35***
HDLc (mg/dL)	52.77 ± 6.68	78.94 ± 3.83**	81.35 ± 5.80**	75.12 ± 3.71**
LDLc (mg/dL)	31.31 ± 2.68	10.16 ± 0.58***	11.03 ± 1.23***	5.51 ± 1.96***
VLDL (mg/dL)	15.16 ± 0.72	18.17 ± 1.08	17.02 ± 1.02	8.92 ± 0.87***
Serum creat (mg/dL)	0.72 ± 0.04	0.63 ± 0.04	0.59 ± 0.02	0.55 ± 0.03**
Urine creat (mg/dL)	0.58 ± 0.03	0.62 ± 0.03	0.66 ± 0.03	0.70 ± 0.04
Creat.CR (mL/min)	3.56 ± 0.24	4.37 ± 0.36	4.93 ± 0.35*	4.95 ± 0.39*
Urea (mg/dL)	47.51 ± 2.61	57.13 ± 3.47	51.34 ± 3.64	41.47 ± 3.13
Uric acid (mg/dL)	13.08 ± 0.79	11.01 ± 0.86	13.53 ± 1.15	12.07 ± 1.03

Values are expressed as mean ± SD, *n* = 10. (One-way ANOVA followed by Turkey’s post-hoc test). *P < 0.05; **P < 0.01; ***P < 0.001 significantly different when compared to the control group

On the other hand, there is a significant decrease in the ALT (*p* < 0.01), AST (*p* < 0.05), triglycerides (*p* < 0.001), VLDL (p < 0.001) and creatinine (*p* < 0.01) at the dose of 250 mg/kg. All doses of CMAE caused a significant decrease (*p* < 0.001) of LDL-c and a significant increase (*p* < 0.01) in HDL-c. In addition, renal clearance has significantly increased (*p* < 0.05) in the blood of animals treated at the dose of 250 mg/kg of CMAE.

### Serum electrolytes concentration

Administration of different doses of extract in rats produced no significant changes in serum electrolyte concentration (sodium, potassium, phosphorus and chloride, compared to the control animals group (Table 7).

**Table 7:** Serum electrolytes concentration of rats after 6 months of treatment with the aqueous extract of *C. molle*

Parameters	Groups			
	Control	62.5 mg/kg	125 mg/kg	250 mg/kg
Sodium (meq/L)	152.58 ± 5.91	154.24 ± 5.87	153.99 ± 7.28	152.42 ± 4.10
Potassium (meq/l)	5.58 ± 0.34	5.45 ± 0.35	5.85 ± 0.33	6.89 ± 0.48
Calcium (meq/L)	11.17 ± 0.41	11.49 ± 0.52	11.43 ± 0.46	14.24 ± 1.37
Chloride (meq/L)	102.59 ± 1.81	102.13 ± 2.30	104.68 ± 1.98	106.78 ± 1.60
Phosphate (mg/dL)	11.39 ± 0.41	12.12 ± 0.55	10.78 ± 0.51	11.61 ± 0.58

Values are expressed as mean ± SD, *n* = 10. (One-way ANOVA followed by Turkey’s post-hoc test)

### Antioxidant parameters

**Fig. 1:**
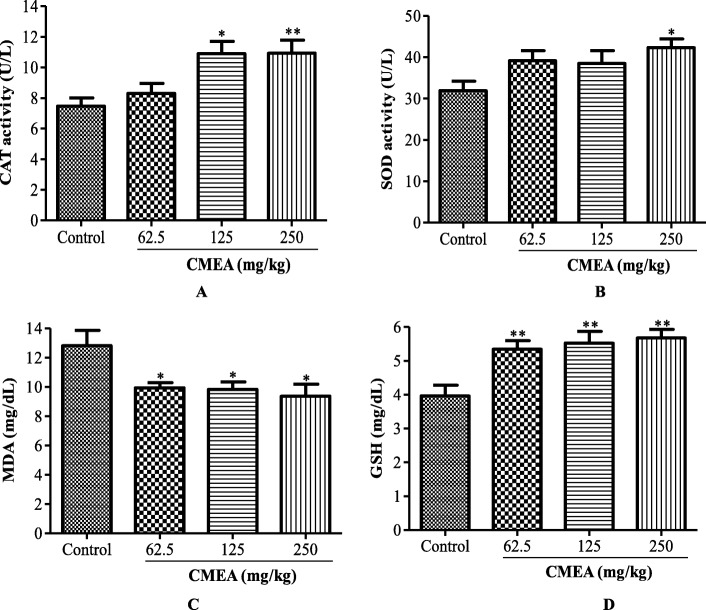
Effect of aqueous extract of *C. molle* on serum antioxidant parameters. **a** Catalase (CAT) activity. **b** Superoxide dismutase (SOD) activity. **c** malondialdehyde (MDA). **d** reduced glutathione (GSH). Values are expressed as mean ± SD, *n* = 10 (One-way ANOVA followed by Turkey’s post-hoc test). *P < 0.05; **P < 0.01; ***P < 0.001 significantly different when compared to the control group

The results show a significant increase in CAT activity at doses of 125 (*p* < 0.05) and 250 mg/kg (*p* < 0.01) as compared to the control group. Only the dose 250 mg/kg of CMAE resulted in a significant increase (*p* < 0.05) of SOD activity. In addition, the GSH rate significantly increased (*p* < 0.01) with all doses of CMAE while the MDA level significantly decreased (*p* < 0.05) in animals treated with various doses of CMAE (Fig. 1).

## Discussion

This study was aimed at evaluating experimentally the chronic toxicity of the CMEA twigs in albino rats. According to the phytochemical screening carried out by Miaffo et al. [6], *C. molle* extract contains the classes of chemical compounds such as flavonoids, saponins, terpenoids and tannins.

General behavior and mortality are the main parameters considered in the evaluation of first signs of toxicity [24]. In this study, there were no observable signs of toxicity and mortality during 6 months of treatment at all the doses used, which is an indication that the extract was well tolerated by the experimental subjects.

The reduction in body weight is considered as the sensitive index of the toxicity of a substance [25]. In the present study, no change in body weight was observed in the subjects throughout the treatment period. Likewise, the extract did not result in any significant change in water and food intake. These results suggest that the extract has no effect on appetite, and consequently on the growth of animals because it has been shown that any factor influencing food intake will also affect body weight.

The change in internal organ weight is an indicator of atrophy and hypertrophy [26]. In this study, the relative weight of the internal organs did not undergo any significant change, suggesting that administration of CMAE twigs at chronic oral doses produces no effect on the normal organ growth.

Assessment of lipid parameters provide useful information on lipid metabolism as well as on the predisposition of animals to atherosclerosis and heart disease [27]. In the present work, the extract resulted in a significant decrease in triglycerides, VLDL and LDL cholesterol and a significant increase in HDLc. These results can be explained by the presence of chemical compounds such as tannins, flavonoids and phenols in the extract which have hypolipidemic, antihyperlipidemic and cardioprotective properties [28]. In addition, flavonoids have been reported to reduce risk-related diseases of the coronary artery [29].

In this study, no change in blood glucose level was observed in subjects throughout the of treatment period. This suggests that the extract had no long-term effect on carbohydrate metabolism.

The levels in total protein, total bilirubin and albumin of the experimental animals showed no significant change; therefore, CMAE has no adverse effect on these parameters.

ALT is a cytosolic enzyme secreted in liver cells and released into the bloodstream by hepatic cell necrosis [30]. It is a liver-specific enzyme, making it an important and very sensitive indicator of hepatotoxicity [31, 32]. AST is also an indicator of hepatocyte destruction, although in addition to the liver it is found in the heart, skeletal muscles, lungs and kidneys [33]. ALP is an abundant enzyme in hepatocyte membranes and used to screen for cholestasis or biliary obstructions [34]. In this study, the concentration of these two enzymes (ALT and AST) decreased significantly in animals treated with doses of 125 and/or 250 mg/kg. These results suggests that the aqueous extract may suggest a potential for hepatoprotective action.

Creatinine, urea, uric acid and renal clearance remain semiotic parameters for diagnosis of renal function [35]. An increase in the level of these parameters in the blood is associated with reduced renal function and increased renal failure. In this study, serum creatinine decreased at the dose of 250 mg/kg and renal clearance of creatinine increased at the dose of 125 and/or 250 mg/kg. CMAE may have nephroprotective potentials and thereby improve renal function.

So far as serum electrolytes is concerned, no significant variation was recorded after administration of the different doses of CMAE for 6 months, which suggested a possible absence of adverse effect of CMAE on ion balance, and consequently on nervous activity.

The evaluation of hematological parameters can be used to determine the extent of the deleterious effect of the extract on the blood [27]. The evaluation of indices of certain blood parameters such as red blood cells, hematocrit, and hemoglobin is of particular importance in the diagnosis of anemia or has harmful effects on erythrocytes. In this study, CMAE induced a significant increase in RBC, HCT, PLT, WBC and Hb levels. Increased rate of erythrocytes, leucocytes and thrombocytes is thought to be due to the overproduction of hematopoiesis (colony stimulating factor, erythropoietin, thrombopoietin) regulatory elements by bone marrow macrophages and strom cells [35, 36]. Moreover, the extract would directly stimulate erythropoiesis, thrombopoiesis and leucopoiesis in the bone marrow [37]. The WBC, lymphocytes, monocytes and granulocytes are mediators of immunity and contribute to the immune protection against inflammation. The increase in the rate of WBC observed is explained by the strengthening of the immune system by CMAE [38].

Malondialdehyde (MDA) is used as markers of lipid peroxidation [39]. Enzymes such as SOD and CAT are part of the defense system against reactive oxygen species [40]. The increase in the level of MDA and the decrease in the activity of antioxidant enzymes imply an imbalance between the production of free radicals and the body’s antioxidant defenses, in favor of the former [41, 42]. In the present work, CMAE has caused a decrease in MDA and an increase in GSH level and antioxidant enzymes (SOD and CAT) activities. This suggests that CMAE has the ability to increase antioxidant defenses in the body and can therefore contribute to the treatment and prevention of degenerative abnormalities due to oxidative stress.

## Conclusions

At the end of this study, no sign of major intoxication associated with treatment with CMAE was noted during 6 months of treatment. In fact, CMAE has no harmful effect on the growth of animals, the weight of internal organs and food and water consumption. CMAE has lipid-lowering, cardioprotective, nephroprotective and hepatoprotective properties. It has the ability to increase the body’s antioxidant defenses and stimulate hematopoiesis to prevent anemia. These results suggest that long-term consumption of CMAE at therapeutic dose (250 mg/kg) presents low risks to human health.

## Data Availability

The datasets used and/or analyzed during the current study are available from the corresponding author upon reasonable request.

## Abbreviations

OECD: Organisation for Economic Co-operation and Development
CMAE: Aqueous extract of *Combretum molle*
CNH: Cameroon National Herbarium
SD: Standard Derivation
ANOVA: Analysis of variance
RBC: Red blood cell
WBC: White blood cells
HCT: Hematocrit
Hb: Hemoglobin
MCV: Mean corpuscular volume
MCCH: Mean corpuscular concentration in hemoglobin
MCH: Mean corpuscular hemoglobin
MPV: Mean platelet volume
ALT: Alanine aminotransferase
ALP: Alkaline phosphatase
AST: Aspartate amino transferase
HDL: High density lipoprotein
LDL: Low density lipoprotein
VLDL: Very low density cholesterol
CR: Renal clearance
SOD: Superoxide dismutase
CAT: Catalase
MDA: Malondialdehyde
GSH: Glutathione
